# Sensory Experience as a Regulator of Structural Plasticity in the Developing Whisker-to-Barrel System

**DOI:** 10.3389/fncel.2021.770453

**Published:** 2021-12-24

**Authors:** Chia-Chien Chen, Joshua C. Brumberg

**Affiliations:** ^1^Department of Psychology, Queens College City University of New York, Flushing, NY, United States; ^2^Department of Neuroscience, Duke Kunshan University, Suzhou, China; ^3^The Biology (Neuroscience) and Psychology (Behavioral and Cognitive Neuroscience) PhD Programs, The Graduate Center, The City University of New York, New York, NY, United States

**Keywords:** microglia, perineuronal net (PNN), barrel cortex, whiskers, develoment

## Abstract

Cellular structures provide the physical foundation for the functionality of the nervous system, and their developmental trajectory can be influenced by the characteristics of the external environment that an organism interacts with. Historical and recent works have determined that sensory experiences, particularly during developmental critical periods, are crucial for information processing in the brain, which in turn profoundly influence neuronal and non-neuronal cortical structures that subsequently impact the animals’ behavioral and cognitive outputs. In this review, we focus on how altering sensory experience influences normal/healthy development of the central nervous system, particularly focusing on the cerebral cortex using the rodent whisker-to-barrel system as an illustrative model. A better understanding of structural plasticity, encompassing multiple aspects such as neuronal, glial, and extra-cellular domains, provides a more integrative view allowing for a deeper appreciation of how all aspects of the brain work together as a whole.

## Introduction

A fundamental question of modern neuroscience centers on how sensory experience shapes the development of the brain and its composite circuitry. Classic works demonstrated that influencing a neocortical neuron’s activity by means of peripheral manipulation such as finger amputation (Merzenich et al., 1984) or dark rearing (Blakemore and Van Sluyters, 1975) can affect many features of neuronal structure and function. Early studies suggested that suturing one eye alters the development of structures within the primary visual cortex (Hubel et al., 1979). Within the somatosensory cortex (S1), studies have found that ablating a whisker follicle at birth prevents the development of that whisker’s cortical representation (Van der Loos and Woolsey, 1973). Within these many models of manipulating sensory-activities a commonality is that early sensory experience, or lack of it, has profound impacts on the developing brain. The rodent whisker-to-barrel system provides a valuable model for exploring this question of experience-dependent plasticity due to its well-defined local circuits, which develop postnatally, and the ease of peripheral manipulation of afferent activity (for review see Erzurumlu and Gaspar, 2020) to this system.

Woolsey and Van Der Loos discovered the cellular conglomerates aggregated in layer 4 of primary somatosensory (S1) in rodents and reported such cellular clusters as “barrels” that represent the contralateral whiskers of the mystacial pad in a one-to-one fashion (Van der Loos and Woolsey, 1973; referred to as S1BF hereafter). They then demonstrated that ablating the vibrissae follicles on the contralateral mystacial pad soon after the rodents’ birth resulted in a large-scale, dramatic reorganization of barrel patterning (Van der Loos and Woolsey, 1973). However, such a large-scale alteration of areal patterning of barrels was reduced following a developmental critical period after the animals have matured (Weller and Johnson, 1975; Woolsey and Wann, 1975) such that after 5 days post birth, the same manipulations no longer impacted the organization of the barrel cortex. The behavioral and physiological consequences of removing vibrissae from birth further suggested that sensory experiences play a critical role in shaping the proper development of neuronal circuitry and its governed behavior (Simons and Land, 1987; Carvell and Simons, 1996). Whisker removal in rodents caused them to no longer distinguish subtle changes in surface texture even after whisker regrowth (Carvell and Simons, 1996), and the neurons corresponding to clipped whiskers become more excitable (Simons and Land, 1987). These seminal works provided the foundation for subsequent research to explore the structural basis of how sensory deprivation affects circuits in the neocortex in general.

**Figure 1:**
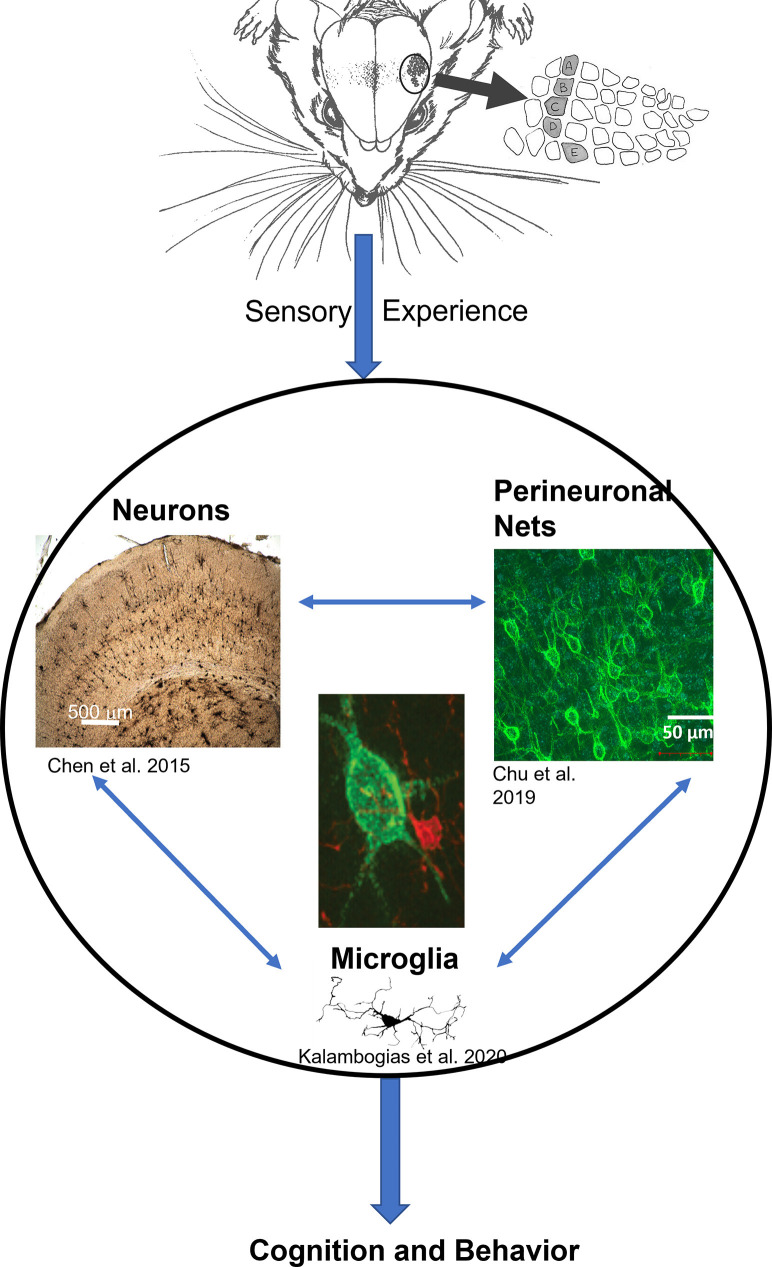
The general model of neuron-glia-ECM triad relationship. Sensory experience conveyed through the animal’s whiskers impacts neuronal morphology (Chen et al., 2012) and spine density (Chen et al., 2015a), at the same time microglia morphology is impacted (Kalambogias et al., 2019) and perineuronal net density is decreased as well (McRae et al., 2007). We hypothesize that changes in sensory experience can influence microglia (center image in red) to interact with the perineuronal net (center image in green) to change their integrity which in turn impacts cellular functioning (Chu et al., 2018). The result would be changes in behavior. Illustration by A. Barrientos.

Over recent years, a branch of research has been focusing on the structural basis of the observed physiological and behavioral plasticity that resulted from chronic sensory deprivation (Zuo et al., 2005; Yang et al., 2009; Briner et al., 2010; Chen et al., 2015a). Such structural basis is not limited to neuronal aspects only but also extends to glial as well as extracellular components as well (McRae et al., 2007; Barrera et al., 2013; Chen et al., 2015b; Chu et al., 2018; Kalambogias et al., 2019), indicating that the impact of chronic sensory deprivation far exceeds the conventional assumption that only neurons are affected. These findings illustrate that multiple systems in the brain are affected simultaneously, further suggesting that neurons, glia, and extracellular components operate in concert with one another, coordinating a symphony that denotes how activities shape the proper development of the brain as a whole. From this multi-faceted perspective, it appears that the extent of experience-dependent structural plasticity in the brain has multiple components, all interacting with one another (see Figure 1), and which may be the structural foundation that ultimately serves as the basis of behavioral and, or cognitive processes.

This review focuses on how sensory deprivation impacts these multiple aspects of extracellular and cellular structure within the brain with a particular emphasis on the S1BF, similar findings have been observed in other sensory systems (Berardi et al., 2003; Persic et al., 2020; Baroncelli and Lunghi, 2021) but are beyond the focus of the current review. In characterizing structural plasticity, researchers traditionally focused on fixed tissue preparations. However, recent advancements in technology have provided new windows of opportunity to peek into the living brain, with the breakthrough of multi-photon *in vivo* imaging techniques. Understanding how cellular structural alterations in general and structural dynamics in specific correlate with anatomical and physiological features of neural circuits is crucial to understanding their role in information processing and functioning in the brain.

## Basic Neuroanatomy of The Whisker-To-Barrel System and Earlier Works of Sensory Deprivation

The barrel cortex is a specialized region of the primary somatosensory cortex (S1) devoted to processing whisker-related information. At the start, the whiskers are perturbed from rest which activates nerve endings embedded in the whisker’s associated follicle, and this tactile based information is transmitted in a mono-synaptic connection through the infraorbital nerve, a sub-portion of the trigeminal nerve, to the trigeminal nuclei, where the first synapse is found. Within the brainstem therein lies the principal nucleus and three spinal nuclei (interpolaris, oralis, and caudalis). The principal nucleus of V (PrV) gives rise to the lemniscal pathway, which targets the ventro-posterior medial (VPM) nucleus of the thalamus on the contralateral side, while the three spinal nuclei give rise to the para-lemniscal pathway, which targets the medial portion of the contralateral posterior thalamic nuclei (POm). From the thalamus, VPM predominantly targets the ipsilateral layers 4 and 6 of the barrel cortical column, while POm innervates layers 1, 2 and 5A (Meyer et al., 2010; Wimmer et al., 2010; Oberlaender et al., 2012; Constantinople and Bruno, 2013; also see Deschênes and Urbain, 2009 for a review). At layer 4 where distinct “barrels” (aggregates of cells) can be observed, VPM innervates the barrel centers, whereas the POm targets the septa, or the inter-barrel space (Wimmer et al., 2010; Oberlaender et al., 2012).

The pioneering work of Van der Loos and Woolsey (1973) was the first to demonstrate how ablation of vibrissae follicles after birth can disrupt the formation of the barrel cortex topographic pattern. However, once the animals develop past a critical period (postnatal day 4), the same follicle ablations no longer impacted barrel pattern formation (Weller and Johnson, 1975; Woolsey and Wann, 1975). Chronically depriving sensory experience after birth impacts many aspects of whisker-to-barrel system, but not the overall topography of the barrel cortex. Behaviorally, it alters whisker-related behavior (Carvell and Simons, 1996) in which after whisker regrowth to full length, animals still cannot discern two different gradients of rough surfaces. Sensory deprivation also alters the physiological properties of barrel cortical neurons (Simons and Land, 1987; Lee et al., 2007), resulting in an enlargement in the size of their receptive field, as well as an increase in the strength of their responses to whisker deflections (Keller and Carlson, 1999; Knott et al., 2002). These earlier works provided a solid foundation that sensory activities are paramount for shaping the cellular environment necessary for the proper functioning of whisker-related behavior and its underlying cerebral circuitries.

## Methodology of Depriving Sensory Inputs on Whisker-To-Barrel System

Sensory deprivation in the whisker-to-barrel system comes in many forms, with the commonality that they impact sensory transduction. We will review the most common means to induce sensory deprivation in the whisker-to-barrel pathway.

### Infraorbital Nerve (ION) Lesion

The ION is a branch of the trigeminal nerve, which gives rise to the starting point of the whisker-to-barrels system. It contains the axons that convey information from the follicle embedded within the mystacial pad into the trigeminal ganglion and then on to the Principal Sensory Nucleus of V and the Spinal Trigeminal nucleus, both located within the caudal brainstem. Peripheral transection of the ION completely blocks the sensory information coming from the mystacial pad to the brainstem, preventing any sensory-related information from reaching higher order processing centers. Hence, this is an extreme form of sensory deprivation. It has been documented that mice which receive ION lesions postnatally (prior to 4 days of age) will not develop the barrel pattern in cortical layer 4. However, ION lesions as a means of sensory deprivation have raised some concerns, as this model has been also used as a model for orofacial pain (Xu et al., 2008), and there is a recent surge of research groups using partial or complete transection of ION as a paradigm to induce neuropathic pain as well as allodynia. This may complicate the validity of ION lesion as a pure form of depriving sensory activities.

### Cauterization

Electrocauterization refers to a surgical technique which utilizes electricity to induce heat to destroy tissue, and in this case, the destruction of whisker follicles located on the mystacial pad. Similar to ION, cauterization is irreversible and has a long-lasting impact on the whisker-to-barrel system. Furthermore, electrocauterization causes skin damage to the mystacial pad, which damages the nerve endings responsible for sensory transmission, thus may complicate the interpretation of “pure sensory deprivation” for the whisker-to-barrel system. Among the most popular uses of electrocauterization is to cauterize one row of whiskers on the mystacial pad, because such manipulation when done early enough in development, causes the corresponding cortical barrels within layer 4 of S1BF to disappear, while the immediately surrounding barrels expand to “fill in” and take their places. However, it has been well documented that this rearrangement of whisker representation within the cortex is developmentally time-sensitive: electrocauterization must be conducted prior to postnatal day 4, otherwise such rearrangement of barrel formation does not occur (Van der Loos and Woolsey, 1973; Belford and Killackey, 1980). This suggests that there is a developmental critical period for peripheral manipulation: after the barrel patterns have formed approximately at postnatal day 4, ION as well as electrocauterization no longer have an impact on the barrel pattern. Both ION and electrocauterization methods may also raise concerns of the validity of micro-level (dendrite morphology, dendritic spines) investigation, as it is not clear to the researchers if the cells being investigated still belong to the macro environment (barrels), as barrel formation is absent, because of dysgranulation of cortical patterning due to such dramatic peripheral manipulations.

### Whisker Trimming

Whisker trimming is considered a milder form of sensory deprivation; some may refer to it as a means of “sensory reduction” rather than sensory deprivation. However, the advantage of whisker trimming over ION or electrocauterization is that trimming does not damage the nerve endings embedded within the mystacial pad. Furthermore, whisker trimming, even from birth, does not disrupt the formation of the barrel cortex pattern in layer 4 of S1BF (McRae et al., 2007; Barrera et al., 2013); this bestows confidence to the researchers that the investigated cells do indeed belong to S1BF. Moreover, the effect of whisker trimming has a profound impact on numerous aspects of neuronal, glial, and extracellular structures. For example, whisker trimming broadens the receptive field and increases neuronal excitability (Simons and Land, 1987; Lee et al., 2007), causes reorganization of dendritic arborizations (Chen et al., 2012), greatly impacts dendritic spine morphology, density, and dynamics (Zuo et al., 2005; Yang et al., 2009; Chen et al., 2015a), decrease perineuronal nets surrounding inhibitory neurons (McRae et al., 2007; Nakamura et al., 2009), and, alters microglia morphology (Kalambogias et al., 2019). Whisker trimming as a means of sensory deprivation has the advantage of reversibility compared to ION lesions and electrocauterization. However, it also has several disadvantages, such as intensive manual labor, due to the constant regrowth of whiskers on the mystacial pad. Another disadvantage of whisker trimming is that starting from approximately when spontaneous movement of the whiskers, whisking, initiates (postnatal day 10–12), mice move their heads frequently as well as spontaneously moving their whiskers, and consequently, require artificial sedation (e.g., isoflurane) in order to trim the whiskers effectively. Furthermore, some delicate trimming methods (e.g., checkerboard trimming, see below) require a more careful and experienced researcher. Overall, whisker trimming requires much more patience compared to ION transection or whisker follicle electrocauterization.

#### Bilateral Trimming vs. Unilateral Trimming

Bilateral trimming refers to trimming all the whiskers on both sides of the face, while unilateral trimming refers to trimming all the whiskers on one side only. Bilateral trimming reduces uniformly all the sensory inputs to both cortical hemispheres, while the unilateral trimming decreases the sensory inputs to the contralateral cortical hemisphere, sparing the ipsilateral sensory input. In the case of unilaterally trimmed animals, some researchers may be tempted to categorize the ipsilateral (the spared side) as the equivalent to the control side, while some do not view them as equal, arguing that trimming one side of the whiskers may cause the rodent to compensate behaviorally and overuse the spared side, leading to increased activities on the spared side (Whitaker et al., 2007). Furthermore, even in the side that corresponds to sensory deprivation, bilateral and unilateral trimming produce different results. For example, it has been documented that both physiology (Popescu and Ebner, 2010) and spine morphologies (Chen et al., 2015a) are slightly different in the affected side following bilateral vs. unilateral trimming, perhaps due to input that is coming from the opposite hemisphere through corpus callosum connectivities (Ramos et al., 2008).

#### Checkerboard Trimming

Checkerboard trimming refers to trimming every other whisker on the mystacial pad, rather than trimming every whisker. Checkerboard trimming is postulated to increase the difference in sensory experience coming from adjacent whiskers and therefore provides competition for the spared whisker to innervate the deprived barrels. This, in turn, produces a novel sensation for the rodents rather than just an overall reduced sensation. Indeed, checkerboard trimming has been shown to increase dendritic spine (an anatomical indicator of excitatory postsynaptic structure) formation over a period of days in the barrel cortex (Fox, 2002; Holtmaat et al., 2006), whereas uniform trimming of whiskers (unilaterally) only decreased spine elimination rate in the contralateral barrel cortex without affecting spine formation rate (Zuo et al., 2005; Yang et al., 2009; Yu et al., 2013; Park et al., 2018).

#### Trimming All but One Whisker

The single-whisker experience (SWE) is achieved by trimming all but one whisker. The affected rodent then must rely solely on sensory information coming from that one spared whisker. This type of novel sensory experience causes Hebbian expansion of the spared corresponding barrel within the S1BF, widening its receptive field (Fox, 2002; Feldman and Brecht, 2005), and enhancing the magnitude of neuronal responses to the deflection of the spared whisker (Glazewski et al., 2007). This is also a popular method for awake behavioral studies that train mice to discriminate different textures while simultaneously performing electrophysiological recordings or optical imaging *in vivo* (O’Connor et al., 2010; Sofroniew et al., 2014; Kwon et al., 2016).

## Effect of Sensory Deprivation on Dendritic Morphology

Neurons come in different shapes and forms, but they all share similar structural subcomponents consisting of soma (cell body), dendrites, and axons. Morphologically, mammalian cortical neurons can be separated into two main categories: pyramidal and non-pyramidal neurons. Pyramidal neurons possess triangular somata, along with apical dendrites that typically project towards the pial surface. Non-pyramidal neurons, in contrast, lack apical dendritic features and are predominantly GABAergic neurons that exhibit smooth dendrites lacking dendritic spines (see White, 1989). Non-pyramidal neurons are extremely heterogeneous, the more common types are basket cells (Somogyi et al., 1983), chandelier cells (Lewis and Lund, 1990), double bouquet cells (Somogyi and Cowey, 1981), Martinotti cells (Fanselow et al., 2008; Xu and Callaway, 2009), neurogliaform (Ferrer et al., 1986), and spiny and aspinous stellate cells (Jones, 1975). Interestingly, morphological heterogeneity of cortical neurons can be found even within a single cortical lamina (Tsiola et al., 2003; Chen et al., 2009). Functionally, pyramidal and spiny stellate cells are the excitatory glutamatergic regular-spiking units (RSUs) in the neocortex (Simons and Land, 1987; McCormick et al., 1985). By contrast, the basket, chandelier, double bouquet, and Martinotti cells are inhibitory GABAergic neurons displaying a variety of physiological properties (Tremblay et al., 2016). Histochemically, neocortical interneurons exhibit a wide range of protein expression, including parvalbumin, somatostatin, 5HT-3a, neuropeptide Y (NPY), vasoactive intestinal polypeptide (VIP), cholecystokinin (CCK), calretinin, and calbindinD28K (Cauli et al., 1997; Lee et al., 2010; Tremblay et al., 2016). How alterations in sensory experience impact specific phenotypes is not well understood, but its differential impact on pyramidal vs. non-pyramidal neurons has been noted (Chen et al., 2012).

In normal development, dendritogenesis follows a simple-to-complex trajectory, in which postnatal neurons exhibit simple structural patterning, and, as development progresses, the dendritic fanning becomes more elaborate (Maravall et al., 2004; Nakazawa et al., 2018). On the other hand, how sensory activities modulate such development of dendritogenesis depends on the duration, onset, and method of sensory deprivation. In general, early onset of sensory deprivation (e.g., before PND4; the time point where the barrel pattern emerges in S1BF) has a more dramatic effect on dendritic fanning than late-onset of sensory deprivation. If sensory deprivation occurs during adulthood or late juvenile developmental stages, it can still cause alterations in other dendritic parameters such as the number and density of spines (see below), it does not lead to dramatic changes in dendritic morphology (Cheetham et al., 2008). For example, trimming whiskers starting at PND 9 led to increased secondary dendritic branching points in layer 2/3 pyramidal neurons’ basilar dendrites, but this effect was lost when trimming commenced at PND 15 (Maravall et al., 2004). Even in the extreme case of electrocauterization in the mature cortex, the overall dendritic length does not appear to be affected (Tailby et al., 2005). Qualitatively, however, the growth polarization pattern of layer 2/3 and layer 4 pyramidal basilar dendrites becomes directed away from the barrel center (Tailby et al., 2005), indicating that sensory activities derived from thalamocortical afferents still play a significant role in guiding the direction of dendritic growth patterns in mature cerebral cortex.

How early developmental (i.e., neonatal) sensory deprivation influences the dendritic morphology seems to be cortical layer specific. Early whisker trimming, even for a short duration such as during just the first 3 days of life, results in a larger dendritic span (area encapsulated by dendrites) in layer 4 spiny stellate cells in S1BF (Lee et al., 2009), consistent with findings following early-stage sciatic nerve transection in gerbils’ S1 layer 3 pyramidal neurons (Macharadze et al., 2019). Interestingly, Sholl analysis (which investigates the complexity of neuronal structures as a function of distance away from the somata; Sholl, 1953) showed a double dissociation relationship: sensory deprived neurons exhibit lower complexity at proximal regions (closer to soma), but higher complexity at distal ending regions. The degree of alteration, however, is greater at distal than proximal, resulting in overall larger dendritic complexity in sensory deprived neurons (Lee et al., 2009). Longer duration of whisker trimming from birth for 1 month also results in expansion of basilar dendrites in layer 6 pyramidal neurons but has the opposite effect on apical dendrites of the same cells, which typically extend to layer 5 and even reach as far as layer 4 in some subgroups (Chen et al., 2009, 2012). The net result is that the total dendritic length remains unchanged. Rather, chronic sensory deprivation leads to a re-distribution of dendritic material, suggesting some sort of structural homeostasis that the neuron is attempting to maintain.

Layer 6 pyramidal cells are not the only neuronal population that show this apical-basilar discrepancy in response to sensory deprivation. Layer 5 pyramidal neurons also share this similarity. Sensory deprivation starting from PND 7 for 1 week leads to lower apical dendritic processes, but higher basilar dendritic processes in Layer 5 pyramidal neurons (Zhang et al., 2013). GABAergic neurons, on the other hand, are less studied in the context of sensory deprivation. Studies have shown that Layer 5 GABAergic neurons exhibit less change in their primary and no change in their secondary dendritic processes following 1 week of whisker trimming (Zhang et al., 2013). By contrast, Layer 6 non-pyramidal neurons, which are presumably GABAergic, have a greater dendritic field and longer dendritic length following 1 month of whisker trimming from birth (Chen et al., 2012). Similarly, sensory deprivation has been shown to change in the density of markers associated with interneurons (Ueno et al., 2015) and as well as a decrease in GABAergic neurons within layer IV (Micheva and Beaulieu, 1995). Within the visual system, sensory loss caused a decrease in spines on a subset of inhibitory interneurons (Keck et al., 2011). Future studies may explore a systematic investigation on how whisker trimming may impact the GABAergic neuronal structure by varying sensory deprivation onset time, duration, and cortical laminae for a comprehensive understanding of how this very critical subset of the neuronal population is influenced by sensory activities. Researchers should avoid a general rule of how sensory activities may impact the development of neurons, because each neuronal class, even in different cortical layers, may respond very differently to the same sensory deprivation method.

## Effect of Sensory Activities on Synaptic Structures

### Effect on Dendritic Spines

Dendritic spines have fascinated generations of neuroscientists since their initial description by Santiago Ramón y Cajal more than a century ago (Ramon y Cajal, 1888). These delicate protrusions emanate from the dendritic shaft and resemble “bristling thorns or short spines” as described by Cajal. They are the postsynaptic sites of the great majority (>90%) of excitatory glutamatergic synapses in the mammalian brain and contain essential molecular components for postsynaptic signaling and plasticity (see Chen et al., 2014). Therefore, spines and their structural dynamics may serve as indicators for synaptic connectivity and modifications thereof (Segal, 2005; Tada and Sheng, 2006; Harms and Dunaevsky, 2007).

**Figure 2:**
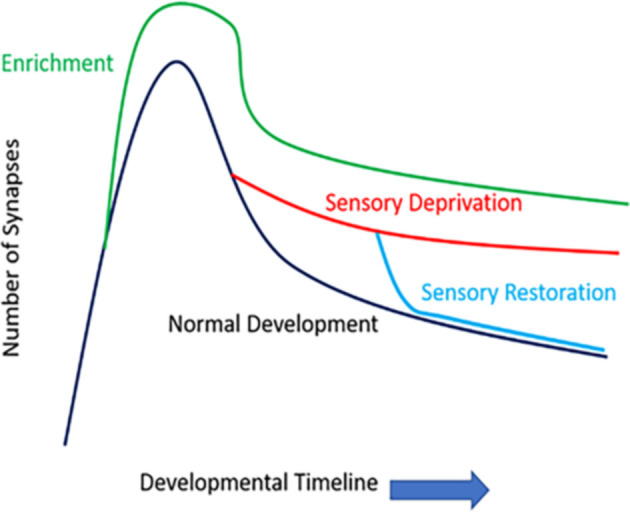
The number of synapses of function development and sensory experience. Rapid spinogenesis in early postnatal is followed by a gradual spine pruning in adolescence. Environmental enrichment generally results in more numerous synapses. Sensory deprivation stunts the synaptic refinement stage, delaying the spine pruning process; whereas restoration of sensory activities can accelerate such activity-dependent spine pruning, even in later developmental stages such as late adolescence.

How sensory information potentially modifies the density and functioning of synaptic structures is widely studied in modern neurobiology. Early works have examined fixed tissue samples (e.g., Golgi stain or electron microscopic level examinations) to observe how sensory information/activity impacts dendritic spines. The densities and morphologies of these dendritic protrusions seem to follow a consistent principle as the brain matures postnatally: there is a general peak in the number and density of dendritic spines (synaptogenesis period), followed by a wane (synaptic pruning period), and both events may be shaped by sensory activities (Figure 2). Overall, sensory/environmental enrichment seems to enhance the growth of dendritic spines (Landers et al., 2011; Jung and Herms, 2014), while sensory deprivation seems to impact synaptic pruning, delaying the maturation of synaptic refinement (Zuo et al., 2005; Chen et al., 2015a). Paradoxically, sensory deprivation and environmental enrichment both lead to increased spine density, but the mechanism of such increase in spine density is most likely different, due to differences in spine elimination and formation rates. Environmental enrichment causes the increased formation of dendritic spines that are in need, while sensory deprivation stunts the elimination of unnecessary spines. Thus, both increased and decreased experience result in increased spine density, which has been demonstrated in fixed-tissue preparations. Furthermore, sensory restoration following deprivation, depending on the age of onset and cortical areas (or layers) investigated, may accelerate the previously stunted synaptic refinement caused by deprivation (Zuo et al., 2005). It has been shown previously in many systems that different sets of dendritic spines likely encode different types of experiences and the storage of memories. For example, in the visual system, when animals re-experienced a second period of monocular deprivation, no additional dendritic remodeling occurred as it did in the first period of monocular deprivation (Hofer et al., 2009). Similarly in the barrel system, when animals re-experienced the same environmental enrichment (EE), there were no additional gain or loss of dendritic spines, but novel EE induced higher spine formation and elimination rates (Yang et al., 2009). In the motor system, animals that were re-trained with a previously learned task showed comparable spine gain/loss rates to controls, but when these animals were trained with a new motor task, their spine remodeling rate once again increased (Xu et al., 2009; Yang et al., 2009). However, it is important to note that in some cases, gaining a behavioral function is not always associated with the formation of new spines, but the elimination of pre-existing spines. For example, in the frontal association cortex, gaining a fear-related memory is associated with the elimination of dendritic spines, while fear extinction is associated with the re-appearance of previously lost spines (Lai et al., 2012). Similarly, in the barrel cortex, acquisition of a tactile variant of trace eyeblink conditioning is associated with the elimination of dendritic spines (Joachimsthaler et al., 2015). Nevertheless, these studies pinpoint that prior experiences leave a lasting trace in the cortical circuits, and specific dendritic segments and their hosted spines likely encode for each specific experience/memory.

Morphologically, dendritic spines come in many shapes and forms (Arellano et al., 2007a, b; Chen et al., 2015a). Overall, they can be categorized into several morphological classes such as mushroom, thin, stubby, branched, as well as some other classes based on morphological appearances (Comery et al., 1997; Irwin et al., 2002; Arellano et al., 2007a; Yu et al., 2013; Chen et al., 2015a; Tjia et al., 2017; Park et al., 2018). Some researchers may also categorize filopodia (thin long headless protrusions) as dendritic spines, while some may disagree since they do not seem to consistently form synapses with presynaptic boutons’ (Arellano et al., 2007b). Nevertheless, morphologies of spines also follow a very consistent principle during development: at younger ages, the brain exhibits higher proportions of “immature” dendritic protrusions consisting of filopodia and thin spines. As animals mature into adulthood, the emergence of “mature” mushroom spines starts to outpace the thin spines and filopodia (Orner et al., 2014; Chen et al., 2015a).

Early studies on the dendritic spine examined fixed neural tissue with light or electron microscopy (Lund et al., 1977; Woolley et al., 1990; Harris and Kater, 1994; Lippman and Dunaevsky, 2005). Although they provided fundamental information about spine morphology and distribution, these fixed tissue examinations only captured static “snapshots” of spines. Over the past decade, fluorescent labeling techniques and multi-photon microscopy enabled time-lapse imaging of dendritic spines in the living brain. A dynamic picture of spines has emerged from such longitudinal studies: spines form, enlarge, shrink, and retract throughout the animal’s lifespan. Furthermore, their morphology and dynamics vary among neuronal types, across developmental stages, and in response to experiences such as sensory stimulation and deprivation, environmental enrichment, and various paradigms of learning (Holtmaat and Svoboda, 2009; Fu and Zuo, 2011; Chen et al., 2014; Joachimsthaler et al., 2015).

Similar to their parent dendrites, dendritic spine dynamics and morphology are impacted by sensory deprivation. During normal development, spine density varies significantly across diverse populations of neurons, likely reflecting the diversity of neuronal morphology and function (Nimchinsky et al., 2002; Ballesteros-Yanez et al., 2006). The balance between spine formation and elimination determines the change in spine density: a surplus of spine formation over elimination along a dendritic segment increases spine density thereon, and *vice versa*. In the cerebral cortex, the rates of spine formation and elimination change over time, resulting in non-monotonic alteration in spine density. For example, spines on the apical dendrites of layer 2/3 pyramidal neurons in the rodent barrel cortex exhibit gradually decreasing motility (elongation and shortening of spines) and turnover rate (defined as the total amount of gains and losses of spines) between postnatal day 7 and 24 (P7–24). Nevertheless, spine density continuously increases over this period of time (Lendvai et al., 2000; Cruz-Martin et al., 2010). After this initial phase of net spine gain, spine elimination starts to outpace formation, leading to an overall reduction of spine density (Zuo et al., 2005; Holtmaat et al., 2006; Yang et al., 2009). Between P28 and P42, 17% of spines are eliminated along the apical dendrites of layer 5 pyramidal neurons in the mouse barrel cortex, while only 5% of new spines are formed during the same time period. Importantly, not all spines are equally susceptible to elimination: those with large heads are more stable than thin ones. As spine head size correlates with synaptic strength, this phenomenon suggests that stronger synapses are more stable (Holtmaat et al., 2006). Furthermore, newly formed spines are more likely to be eliminated than pre-existing spines (Xu et al., 2009), and the majority of stable spines formed before adolescence remain incorporated in the adult neuronal circuit (Zuo et al., 2005; Yang et al., 2009; Yu et al., 2013). Finally, in adult animals, spine formation and elimination reach equilibrium; spine density remains roughly constant until the onset of aging (Zuo et al., 2005; Mostany et al., 2013).

During the early postnatal period, sensory inputs play instructive roles in the stabilization and maturation of spines. In the mouse visual cortex, depriving visual input prevented the decrease in spine motility and maturation of spine morphology (Majewska and Sur, 2003; Tropea et al., 2010). In mice that had been subjected to visual deprivation previously, light-induced spine maturation could be partially mimicked by pharmacological activation of the GABAergic system, suggesting an important role of inhibitory circuits in the maturation of excitatory synapses (Tropea et al., 2010). Later, sensory experience drives spine pruning (defined as the net loss of spines). Unilateral trimming of all whiskers in 1-month-old mice for 4 or 14 days dramatically reduced spine elimination in the barrel cortex but left spine formation largely unperturbed (Zuo et al., 2005; Yu et al., 2013). However, pyramidal neurons from different layers may respond differently to the same whisker-trimming manipulation (Tjia et al., 2017). Pharmacological blockade of NMDA receptors mimicked the effect of whisker trimming, indicating the involvement of the NMDA receptor pathway in such activity-dependent spine elimination (Zuo et al., 2005). In addition, astrocytes have been shown to participate in spine pruning by influencing synaptic glutamate uptake (Yu et al., 2013).

While complete whisker trimming removes sensory input globally, trimming every other whisker (“checkerboard trimming”) presumably amplifies any difference in activity levels and patterns of neighboring barrels, thereby introducing a novel sensory experience. Such paradigm has been shown to promote spine turnover and to stabilize newly formed spines selectively in a subclass of cortical neurons (Trachtenberg et al., 2002; Holtmaat et al., 2006). New spines were preferentially added onto layer 5 pyramidal neurons with complex apical tufts, rather than those with simple tufts (Holtmaat et al., 2006). Analogously, brief monocular deprivation (MD) increases the disparity between the inputs from two eyes. Thus, similar to checkerboard trimming, MD has been found to increase spine formation along the apical dendritic tufts of layer 5 pyramidal neurons in the binocular zone of the mouse visual cortex. However, this effect was not observed in layer 2/3 neurons, or in the monocular zone (Hofer et al., 2009), again indicating a cell type-specific and layer-specific synapse remodeling similar to the barrel system (Tjia et al., 2017). Interestingly, a second MD failed to increase spine formation further, but selectively enlarged the spines formed during the initial MD, suggesting that new spines formed during the initial MD had functional synapses that were reactivated during the second MD (Hofer et al., 2009).

While the exact molecular mechanisms of sensory deprivation induced alterations of spine dynamics remain elusive, there are several studies providing potential clues. For example, Retinoid acid (RA), which is involved in transcriptional regulation of neurodevelopmental processes mediated by nuclear RA receptors, seems to regulate dendritic spine dynamics. Transcranial two-photon imaging revealed a significant increase in dendritic spine elimination on apical dendrites of somatosensory cortical layer 5 pyramidal neurons in these mice. Interestingly, the enhancement of spine elimination is experience-dependent as whisker trimming rescued the spine elimination phenotype. Furthermore, the conditional KO mice that lacked RA exhibited increased elimination of mature mushroom spines, in which whisker trimming was rescued (Park et al., 2018). These findings suggest that RA may be more preferentially expressed on these mature, stable types of spines. Another molecular mechanism that is involved in sensory development are ephrins, which are guidance molecules. Particularly, the ephrin A2 receptor seems to play a pivotal role in stabilizing existing dendritic spines, as the ephrin A2 KO mice exhibited accelerated dendritic spine elimination in the barrel cortex, but ephrin A3 KO mice exhibited normal spine dynamics. Whisker trimming in ephrin A2 KO mice, similar to the RA KO mice, also rescued this increased spine elimination phenotype. In addition, this mechanism seems to be NMDA-receptor mediated, because MK801 (non-competitive blocker of NMDA-R) *in vivo* injection can also rescue such accelerated spine elimination phenotype in ephrin A2 KO mice (Yu et al., 2013). Unlike the RA KO mice that exhibited preferential elimination of mushroom spines, ephrin A2 KO mice preferentially eliminated the thin spines instead. This suggests that different spine morphologies may harbor specific types of molecular signals/receptors. Fragile X mental retardation protein (FMRP) may also play a role in sensory-experience-dependent plasticity of dendritic spines. Non-deprived *Fmr1* (the gene responsible for producing FMRP) KO mice exhibits both higher dendritic spine formation and elimination (Pan et al., 2010). Normally, 2 weeks of trimming stunts spine elimination while not affecting formation in WT mice, in contrast, spine elimination rates are not impacted by trimming in *Fmr1* KO mice, as both spine elimination and formation remain high in the KO mice even in whisker-trimmed conditions. Lastly, a recent study found that layer 2/3 pyramidal neurons’ basilar dendrites respond functionally to chessboard trimming, but not to all-whisker deprivation, by increasing the production of new dendritic spines. These experience-dependent plasticities were absent in αCaMKII70 T286A mutants that lack LTP, suggesting αCaMKII auto-phosphorylation is an important element for the enlargement of spines, production of new spines, and LTP, thus providing a tighter link between the structural and physiological plasticity (Seaton et al., 2020). As transcranial 2-photon microscope is gaining popularity in the neuroscience field, in addition to the advancement of transgenic mouse technologies, there will be more valuable studies like these to help us gain a more complete knowledge regarding the mechanisms that are responsible for experience-dependent plasticities of dendritic spines in the living brain.

### Effect on Axons and Boutons

Sensory experience does not only impact dendritic and spine development but also impacts the other aspects of the neuron: the soma, its axonal branches, and boutons. Developmentally, the dynamics of axonal boutons are somewhat similar to that of dendritic spines, in which young mice exhibit higher turnover rates and higher net-loss of boutons than adult mice, and these boutons are stabilized during adulthood (Qiao et al., 2016). Whisker trimming in rats from PND 7 for 2 weeks resulted in a reduction of overall axonal length of layer 2/3 to layer 5 corticocortical connections in rats (Bruno et al., 2009). Thalamocortical (TC) afferents in adult rats for 13–27 days also resulted in the reduction of overall axonal length and TC synapses but did not alter the density of TC synapses. Partial whisker trimming (trimming either the upper or lower rows) starting from PND 19 for 13–41 days results in strengthening of the axonal components in the spared cortex when compared to the control cortex. Specifically, the size of axonal varicosities, as well as the contact zones (between boutons and spines), were larger in the spared cortex. In the same partial deprivation paradigm, there is also a rapid reorganization of the axons in both excitatory and inhibitory cells, with a transient increase in axonal bouton density. In the horizontally projecting axons (e.g., layer 2/3 to layer 2/3 connections) there is a net increase of axonal projections from non-deprived whisker barrel columns into the deprived barrel columns. The axons from the inhibitory neurons located in the deprived whisker barrel over-reached in their long–range projections towards the non-deprived whisker barrel columns (Marik et al., 2010). In a separate study using a similar partial sensory deprivation paradigm, it was found that the axonal boutons corresponding to the spared whiskers exhibited larger volume than trimmed whiskers. Furthermore, the bouton volume augmentation occurs only in the en-passant boutons but not terminal boutons. Most importantly, it was found that the sensory-deprived synapses are much more unreliable in which they exhibited significantly higher failure probability to elicit an action potential. Other key physiological features of these deprived synapses are decreased maximal inducible EPSP, correlating with decreased volume of dendritic spine heads (Cheetham et al., 2014). Lastly, sensory activities can also drive homeostatic plasticity in the axonal initial segments: long-term sensory deprivation-induced length increase of axonal initial segments, accompanied with an increase in neuronal excitability, while enrichment caused the axonal initial segment to shorten, with an accompanying decrease in action potentials (Jamann et al., 2021).

## Effect of Sensory Activities on Extracellular Components

The formation, stabilization, and refinement of CNS synaptic connections involves the complex interplay between the developing neuronal cell surface and the molecules in the extracellular space. Perhaps, one of the most influential components is the extracellular matrix (ECM), which is thought to play the role of the scaffolding, or the “glue” that supports the cellular structural frame. The perineuronal net (PNN), a neuron-specific type of ECM, is often found ensheathing cortical parvalbumin (PV)-positive inhibitory neurons and their proximal dendrites in a lattice-like structure and has been postulated to play important roles in neural development, regulation of plasticity, as well as the proper functioning of the CNS (Hockfield et al., 1990; Celio and Blümcke, 1994). There are many subcomponents of the PNNs, including aggrecan, hyaluronan, neurocan, versican, brevican, phosphacan, and chondroitin sulfate proteoglycans (CSPGs). The detailed molecular composition of the ECM is out of the scope of this review (for detailed reference see Sorg et al., 2016; Fawcett et al., 2019). The distribution in the barrel cortex for these subcomponents is not uniform. Rather, the immunoreactivity of chondroitin-6-sulfate containing proteoglycan (CS-6-PG), phosphacan, and neurocan are stronger at barrel septa as compared with barrel hollows and surrounding cortex, while the labeling of Wisteria floribunda agglutinin (WFA) was observed to be strongest in the barrel hollows (Nakamura et al., 2009). In the adult visual system, degradation of the CSPGs has been shown to increase dendritic spine motility as little as 3 h *in vivo* (de Vivo et al., 2013), consistent with previously observed in CA1 neurons *via* organotypic hippocampal slices preparation (Orlando et al., 2012), suggesting that CSPGs restricts morphological changes of synapses. Whether these effects may be generalized to the whisker-to-barrel system is yet to be determined. Following whisker trimming from neonates in both mice and rats, there is a reduction in aggrecan expression and WFA expression (McRae et al., 2007), with the WFA expression in the septa being unaltered (Nakamura et al., 2009). Univibrissa rearing (trimming all but one whisker) resulted in an increase of PNN density in the deprived barrels, but only those that immediately neighbor the non-deprived barrel. Although checkerboard pattern trimming impacts dendritic spines, surprisingly, it had no effect on the density of the PNNs (Nowicka et al., 2009). Overall, sensory experiences seem to regulate the formation of ECM, particularly PNNs, which directly impacts the maturation profile of PV+ interneurons, a critical player of neurodevelopment in the cerebral cortex.

There are other components that can impact the extracellular matrix as well. One such well-studied component is the serine protease tissue-type plasminogen activator (tPA). tPA is a well-known treatment that dissolves blood clots in stroke patients. However, it is also present in the nervous system endogenously. In the visual system, tPA is developmentally regulated, with its levels peaking around the critical period, and decreasing progressively into adulthood (Zheng et al., 2008). Furthermore, tPA levels are also activity-dependent; monocular deprivation spanning the developmental critical period is associated with a significant increase in tPA activity, and such increase is regulated by glutamate decarboxylase-65 (GAD65; Mataga et al., 2002). Mice lacking the gene encoded for GAD65 (GAD65-KO) failed to show such monocular deprivation-induced increase of tPA activities. Additionally, tPA has also been implicated in the regulation of synaptic plasticity. Application of tPA accelerates the dynamics of dendritic spines (Oray et al., 2004), whereas the genetic deletion of tPA prevented monocular deprivation-induced changes of dendritic spine density in the visual cortex (Mataga et al., 2004), and prevented stress-induced loss of dendritic spines in the hippocampus (Pawlak et al., 2005). In the barrel system, it has been shown that tPA is present in microglia, excitatory neurons, and parvalbumin+ neurons, while absent in GIN+ somatostatin interneurons (Chu et al., 2015). A month of whisker trimming results in elevated tPA expression in all layers of the barrel cortex, with some subtle difference between bilateral vs. unilateral trimming in the affected contralateral side (Chen et al., 2015b). Interestingly, in the spared barrel cortex (ipsilateral to the trimmed side), tPA expression is decreased compared to the control animals. Such change may be attributed to mice using the spared whiskers significantly more, compensating for not having one side of the whiskers. So far, it is not known if the changed level of tPA is the reason that PNNs are impacted by sensory deprivation, or if there are other extracellular matrix factors such as matrix metalloproteinase-9 (MMP-9) that may partake in such regulation of sensory-deprivation induced PNN changes, as recently demonstrated in the auditory cortex (Wen et al., 2018). It may be especially rewarding for future studies to systematically explore such possibilities, in order to further elucidate the mechanisms behind sensory activity induced PNN alterations.

## Microglia Response to Changes in Sensory Activities

Microglia are the central nervous system’s principal immune cells which are mostly derived from the mesodermal yolk sac in addition to a few specific circumstances in which microglia can be derived from bone marrow. Microglia are primarily self-sustained through cell division with an average renewal rate of 4 years (Réu et al., 2017). In the traditional view, the primary function of microglia is to protect and maintain CNS integrity (Banati and Graeber, 1994), which is mediated through surveying the extracellular environment and scavenging for foreign bodies or injured tissue (Kreutzberg, 1996; Batchelor et al., 1999; Aloisi, 2001), phagocytosis (Morsch et al., 2015), antigen presentation (Gehrmann et al., 1995; Aloisi et al., 1998; Aloisi, 2001) and cytotoxicity (Banati and Graeber, 1994; Gehrmann et al., 1995; Medzhitov and Janeway, 2000). While microglia were classically thought to be a homogeneous population with little function in the healthy brain, recent work indicates that they are a largely heterogeneous population (Hammond et al., 2019) which dynamically responds to changes in the extracellular environment. For example, in contrast to homeostatic conditions in which microglia exhibit a complex and ramified morphology, during brain pathogenesis, microglia have been shown to exhibit an altered phagocytic-like phenotype (Morsch et al., 2015). Recent work has continued to elucidate the morphological distinctions in microglial morphology in response to various environmental stimuli. Moreover, human research into aging-associated brain dysfunctions has indicated alterations in microglial morphology (Mosher and Wyss-Coray, 2014; Bachstetter et al., 2015), but interestingly, dystrophic microglial morphology are not associated with healthy aging in humans (Shahidehpour et al., 2021). Taken together, these studies provide evidence to suggest that morphological changes in microglia can be used as a proxy to assess associated changes in the micro-environment of the brain.

Over recent years, electron microscopy work assessing the role of microglia within focal cortical inflammation found an increase in microglial density around the legion site and observed that microglial processes make direct contact with neuronal perikaryal and apical dendrites, which was followed by the displacement of a large portion of axosomatic synapses (Trapp et al., 2007). This microglia-neuron interaction indicated a potential role of microglia in neuroplasticity which was later supported by research showing that microglia exhibit experience-dependent participation of the elimination of postsynaptic structures in the visual system (Tremblay et al., 2010). In the motor cortex, microglia depletion *via* diphtheria toxin administration caused a reduction of motor-learning- related dendritic spine remodeling, associated with deflective motor learning skills (Parkhurst et al., 2013). In developing primary somatosensory cortex, both ablation of microglia and minocycline (inhibitor of microglia) administration led to a decreased dendritic spine density with accompanied reduction of mEPSC frequency, without affecting the amplitude (Miyamoto et al., 2016).

In the barrel system, there is some evidence that microglia respond to sensory deprivation. It is known that while chronic sensory deprivation does not alter the cellular density of microglia in the barrel cortex, it dramatically alters the morphological characteristics of microglia. Following sensory deprivation, somatic size is greatly increased while the number of microglial processes and their length are reduced, indicating a morphological shift from the ramified state towards a more “activated” state (Kalambogias et al., 2019). The increase in somatic size may be evidence of increased engulfment of synaptic components by the microglial lysosome, such as vesicular glutamate transporter 2 (vGlut2), a presynaptic component of thalamocortical (TC) afferents (Gunner et al., 2019). Furthermore, it is known that microglia also participate in whisker lesion-induced synapse elimination, as knock out mice lacking the microglial fractalkine receptor, CX3CR1, were not affected by whisker lesions. *ADAM10*, a metalloprotease known to cleave CX3CL1 (ligand for CX3CR1) into a secreted form, is increased specifically in layer IV neurons and microglia following cauterization. Indeed, pharmacological inhibition of ADAM10 successfully blocked the effect of whisker-lesioning-induced TC elimination in the barrel cortex (Gunner et al., 2019). This suggests microglia not only respond to changes in sensory experience but also play a role in dendritic and synaptic remodeling. The direction of microglia-neuronal communication in the context of sensory activity remains unclear. However, it is known that microglia express glutamate receptors including AMPA, NMDA, and mGluRs (Noda et al., 2000; Taylor et al., 2002, 2003; Byrnes et al., 2009; Murugan et al., 2011; for a review see Liu et al., 2016) as well as GABA receptors (Kuhn et al., 2004; Mead et al., 2012), and the activation/inhibition of these receptors on microglia can directly influence their morphological phenotype (for a review see Liu et al., 2016). It is known that the density of GABA-immunoreactive cells is reduced following chronic whisker trimming (Micheva and Beaulieu, 1995), so this decreased GABA expression in the barrel cortex may be responsible for the altered morphological phenotype observed in the chronically deprived animals. It may be rewarding for future studies to systematically explore the bi-directional nature of microglia-neuronal communication, perhaps through the use of specific Cre-lines and chemogenetic/optogenetic methods.

## Astrocytic Response to Changes in Sensory Activities

Astrocytes are a subtype of glial cells within the nervous system. Our understanding of the role astrocytes play has evolved from the traditional supportive cells of neurons to a key player of brain functioning, and may play important roles in neuropathologies (for reviews see Santello et al., 2019; Siracusa et al., 2019). Astrocytes participate in synaptic functioning, particularly with glutamate signaling, forming the “tripartite synapse” consisting of the pre-synaptic axonal bouton, post-synaptic dendrite/spine, and peri-synaptic astrocytic process (Araque, 1999). It has been demonstrated in the visual system that astrocytes are influential in closing the developmental critical period, and transplanting immature astrocytes into an adult cortex reopens the critical period in the visual system, and this process is guided by connexin-30, a subunit of gap junction channels. It was shown that astroglial connexin-30 works to inhibit the expression of MMP-9 (matrix metalloproteinase-9) *via* the RhoA-ROCK (Rho-associated coiled-coil-containing protein kinase 2) pathway (Ribot et al., 2021). Also in the visual system, both monocular and binocular deprivation induced alterations of astrocytes, in which binocular deprivation showed a stronger impact on reactive astrocytes, while concomitantly strengthening the gap junctions between astrocytes in V1 (Wang et al., 2021). These findings suggest that astrocytes may work in concert with neuronal activity in order to shape the circuit remodeling of the brain. Interestingly, in the visual cortex of rats exposed to dark-rearing, there were lower expression levels of S100beta (a calcium-binding protein primarily expressed in a subset of astrocytes; Argandona et al., 2009). Such a reduced level of S100beta can be partially rescued by increased physical exercise, and full recovery can be induced by multisensory environmental enrichment (Bengoetxea et al., 2013) suggesting that astrocytes are influenced by neuronal activity. In the whisker-to-barrel system, a very limited number of published work is available on the effect of sensory deprivation on astrocytes. It is known that whisker deprivation by means of electrocauterization does not change the density of Sox2+GFAP+ radial-glia-like astrocytes (Gonzalez-Perez et al., 2018), which is consistent with the previous finding that chronic whisker trimming from birth does not impact the overall number of Nissl+ cells in barrel cortex (Barrera et al., 2013). However, gross measurements such as cell counts may not be a sensitive enough means to decipher the fine nuances of microcellular alteration on a synaptic level, which is most likely where and how the astrocytes are being modified following prolonged changes in sensory activities. Future studies may emphasize on this particularly unknown area of neuroplasticity. Other glial components may also play a role in circuit reorganization following sensory deprivation (e.g., Barrera et al., 2013), but are beyond the present discussion.

## Future Directions

Sensory activities impact multiple structures at the cellular level within the brain, including neuronal, glial, and extracellular components. As detailed above, sensory deprivation has been shown to impact dendrites and their associated spines in the whisker-to-barrel system. Similar observations have been made in the visual (Hofer et al., 2009) and auditory (Clemo et al., 2016) systems wherein prolonged sensory deprivation alters the underlying circuitry of the primary sensory cortex. Given the easy ability to target specific areas (whiskers) for sensory deprivation (or enrichment), the insights gained from the whisker-to-barrel cortex system can help inform the mechanism(s) that sensory deprivation engages to alter cortical circuitry more generally.

Although these studies have significantly advanced our understanding of how sensory experiences may be the initial trigger of structural plasticity, many questions remain on various fronts. In essence, the interactions of neurons, glia, and ECM in this context are a derivation of the familiar reframe of what came first: the chicken or the egg? For example, in the complex interplay of neuronal-glial-ECM interactions, which alteration following sensory experience/deprivation occurs the earliest? Are all these changes happening simultaneously, or is there a “leading” cause that the other components are compensating for? Alternatively, does one component serve as a permissive role for another to function properly? It is worth noting that almost all works discussed here have focused on a uni-readout approach, i.e., investigating only one dependent variable at a time. Future studies should focus on technical manipulation of neuronal/glial/ECM activities in combination with sensory experience alterations to gain a clearer perspective on how one domain (e.g., neuronal) may influence other domains (e.g., glial/ECM). With technological advances of chemo/optogenetic control of neuronal and/or glial activities, as well as ECM/glial mouse KO/I models, the manifestations of such studies may soon be on the horizon, thus will provide further elucidation of the cellular mechanisms governing experience-dependent plasticities in the brain.

## Author Contributions

C-CC and JB contributed equally to this work, discussed concepts, wrote and edited in tandem. All authors contributed to the article and approved the submitted version.

## Conflict of Interest

The authors declare that the research was conducted in the absence of any commercial or financial relationships that could be construed as a potential conflict of interest.

## Publisher’s Note

All claims expressed in this article are solely those of the authors and do not necessarily represent those of their affiliated organizations, or those of the publisher, the editors and the reviewers. Any product that may be evaluated in this article, or claim that may be made by its manufacturer, is not guaranteed or endorsed by the publisher.
